# A Novel Biomimetic Tool for Assessing Vitamin K Status Based on Molecularly Imprinted Polymers

**DOI:** 10.3390/nu10060751

**Published:** 2018-06-11

**Authors:** Kasper Eersels, Hanne Diliën, Joseph W. Lowdon, Erik Steen Redeker, Renato Rogosic, Benjamin Heidt, Marloes Peeters, Peter Cornelis, Petra Lux, Chris P. Reutelingsperger, Leon J. Schurgers, Thomas J. Cleij, Bart van Grinsven

**Affiliations:** 1 Maastricht Science Programme, Faculty of Science and Engineering, Maastricht University P.O. Box 616, 6200 MD Maastricht, The Netherlands; kasper.eersels@maastrichtuniversity.nl (K.E.); hanne.dilien@maastrichtuniversity.nl (H.D.); joe.lowdon@maastrichtuniversity.nl (J.W.L.); erik.steenredeker@maastrichtuniversity.nl (E.S.R.); renato.rogosic@maastrichtuniversity.nl (R.R.); benjamin.heidt@maastrichtuniversity.nl (B.H.); thomas.cleij@maastrichtuniversity.nl (T.J.C.); 2 Division of Chemistry and Environmental Science, School of Science and the Environment, Faculty of Science and Engineering, Manchester Metropolitan University, Chester Street, Manchester M1 5GD, UK; m.peeters@mmu.ac.uk; 3 Soft-Matter Physics and Biophysics Section, Department of Physics and Astronomy, KU Leuven, Celestijnenlaan 200 D, B-3001 Leuven, Belgium; peter.cornelis@kuleuven.be; 4 Department of Biochemistry, Cardiovascular Research Institute Maastricht, Universiteitssingel 50, 6200 MD Maastricht, The Netherlands; p.lux@maastrichtuniversity.nl (P.L.); c.reutelingsperger@maastrichtuniversity.nl (C.P.R.); l.schurgers@maastrichtuniversity.nl (L.J.S.)

**Keywords:** thermal biosensor, vitamin K, synthetic receptors, HPLC validation

## Abstract

Vitamin K was originally discovered as a cofactor required to activate clotting factors and has recently been shown to play a key role in the regulation of soft tissue calcification. This property of vitamin K has led to an increased interest in novel methods for accurate vitamin K detection. Molecularly Imprinted Polymers (MIPs) could offer a solution, as they have been used as synthetic receptors in a large variety of biomimetic sensors for the detection of similar molecules over the past few decades, because of their robust nature and remarkable selectivity. In this article, the authors introduce a novel imprinting approach to create a MIP that is able to selectively rebind vitamin K_1_. As the native structure of the vitamin does not allow for imprinting, an alternative imprinting strategy was developed, using the synthetic compound menadione (vitamin K_3_) as a template. Target rebinding was analyzed by means of UV-visible (UV-VIS) spectroscopy and two custom-made thermal readout techniques. This analysis reveals that the MIP-based sensor reacts to an increasing concentration of both menadione and vitamin K_1_. The Limit of Detection (LoD) for both compounds was established at 700 nM for the Heat Transfer Method (HTM), while the optimized readout approach, Thermal Wave Transport Analysis (TWTA), displayed an increased sensitivity with a LoD of 200 nM. The sensor seems to react to a lesser extent to Vitamin E, the analogue under study. To further demonstrate its potential application in biochemical research, the sensor was used to measure the absorption of vitamin K in blood serum after taking vitamin K supplements. By employing a gradual enrichment strategy, the sensor was able to detect the difference between baseline and peak absorption samples and was able to quantify the vitamin K concentration in good agreement with a validation experiment using High-Performance Liquid Chromatography (HPLC). In this way, the authors provide a first proof of principle for a low-cost, straightforward, and label-free vitamin K sensor.

## 1. Introduction

Synthetic receptors such as Molecularly Imprinted Polymers (MIPs) have gained interest in recent years, since these plastic antibodies mimic the affinity a natural receptor has for its target but overcome most of the disadvantages associated with their use [1,2]. Traditionally, MIPs were designed to act as a stationary phase in a wide variety of applications for affinity separation, utilizing the remarkable selectivity of these polymeric receptors to purify a compound of interest from a complex mixture [3,4,5,6]. In addition, MIPs have grown in popularity for their use as synthetic receptors in biomimetic sensors, owing to their low-cost and straightforward synthesis process [7], their unlimited shelf life [8], their chemical and thermal stability [9], and their robust nature [10]. 

Although MIP-based sensors for point-of-care diagnostics are not yet ready for the market, interesting progress is made in this field. MIPs have been combined with electrochemical transducers such as amperometry [11,12], cyclic voltammetry [13,14], potentiometry [15,16], or impedance spectroscopy [17,18,19] in a wide variety of sensor platforms. Alternative readout alternatives include optical or gravimetrical methods [20,21,22,23]. In 2012, the authors of this article introduced a thermal readout platform that monitors the thermal resistance of a chip, allowing for the identification of point mutations in DNA [24,25]. Over the years, this versatile transducer has been combined with various natural and biological receptors for the detection of cancer cells [26,27], bacteria [28,29], and proteins [30]. However, the combination with MIPs for the detection of small molecules has proven to be particularly interesting [31,32]. In a previous study, the thermal readout methodology has been improved by analyzing the propagation of a thermal wave rather than monitoring the thermal resistance at a constant input temperature, resulting in an improved sensitivity and reaction time [29,33].

In this work, the authors have incorporated MIPs into a thermal biomimetic sensor platform for the selective detection of vitamin K in hexane-extracted blood serum samples. Vitamin K is very interesting from a medical point of view, as it plays an essential role in blood coagulation and regulates the calcification of bones, blood vessels, and other soft tissue [34]. Additionally, it functions as a co-factor in the post-translational carboxylation of several coagulation factors and regulatory proteins such as Matrix Gla Protein (MGP) and osteocalcin that regulate the mineralization of vascular tissue and bone respectively [34,35,36]. Phylloquinone or vitamin K_1_ is the primary dietary form of vitamin K found in nature [37]. In adults, vitamin K_1_ can be converted into vitamin K_2_, a process that requires the intermediate vitamin K_3_ (menadione) [38]. Vitamin K_2_ accumulates in extrahepatic tissues, whereas K_1_ is the major hepatic form stored in the body [38]. As the concentration of phylloquinone in breast milk and the hepatic vitamin K reserve are low, breast-fed babies are at risk of developing vitamin K deficiency, leading to Vitamin K Deficiency Bleeding (VKDB) which can then lead to brain injury, death, or lifelong impairment [39]. This condition can be prevented by providing supplementary vitamin K_1_ to neonates. In adults, clinical vitamin K deficiency is rare as it is abundantly present in the human diet. However, in some cases (e.g., in patients suffering from cystic fibrosis liver damage, people undergoing dialysis) deficiency can arise [40,41,42,43,44], and low levels of vitamin K are associated with coagulopathy [45], osteoporosis [46], and coronary heart disease [47]. Accurately determining vitamin K status is costly, difficult, and often based on indirect tests that measure the amount of uncarboxylated osteocalcin, uncarboxylated MGP, or antagonism factor-II (PIVKA-II). Additionally, these tests are often based on laboratory techniques such as Enzyme-Linked Immuno Sorbent Assays (ELISA) [42,48], which require labeling. Direct measurements of fasting phylloquinone concentrations in plasma can be performed using reverse-phase liquid chromatography, a technique that requires expensive instrumentation, trained operators and a lab environment [49]. Therefore, a low-cost, label-free test that can determine the amount of vitamin K directly would be of great interest and help to the medical community. 

The experiments described in this article provide a proof of concept for such a sensor platform. Methacrylic acid-based polymers were imprinted with the synthetic vitamin K compound menadione (vitamin K_3_). The structure of vitamin K_3_ is identical to that of the head group of its natural counterpart, but it lacks the long aliphatic side chain that would complicate imprinting. The authors hypothesized that the binding sites within the MIPs would be formed upon interaction with functional groups in the head group of the molecule, and the menadione-imprinted MIP should therefore be able to bind both the synthetic and the natural vitamin K. The thermal analysis of the rebinding capacity of the MIPs confirms this hypothesis. The results obtained with non-imprinted reference electrodes and a study using vitamin E as an analogue indicate that rebinding is both selective and specific. The response was successfully quantified, and a detection limit of 200 nM was established. In addition, the sensor was able to detect vitamin K in hexane-extracted blood serum samples drawn from one of the authors before and after ingestion of vitamin K-rich supplements. A progressive enrichment strategy was chosen to overcome the apparent limiting sensitivity of the readout technique. 

## 2. Materials and Methods

### 2.1. Chemicals

Methacrylic Acid (MAA), chloroform, and aluminum oxide were purchased from Acros Organics. Methanol absolute, acetic acid, and toluene were bought from Biosolve Chimie SARL (Valkenswaar, The Netherlands). Phylloquinone (vitamin K_1_), PolyVinylChloride (PVC), and Menadione (vitamin K_3_) were supplied by Sigma-Aldrich (Zwijndrecht, The Netherlands). Acetone pure and Phosphate-Buffered Saline (PBS) tablets were purchased from VWR chemicals (Amsterdam, The Netherlands). Ethylene Glycol DiMethAcrylate (EGDMA) was procured from Merck Schuchardt OHG (Hohenbrunn, Germany). dl-α-Tocopherol, 97+% (Vitamin E) was supplied by Alfa Aesar (Karlsruhe, Germany). PolyDiMethySiloxane (PDMS) stamps were made with the Sylgard 184 elastomer kit from Mavom NV (Kontich, Belgium). Aluminum chips were purchased at Brico NV (Korbeek-Lo, Belgium) and cut to the desired dimensions.

### 2.2. MIP Synthesis

**Figure 1:**
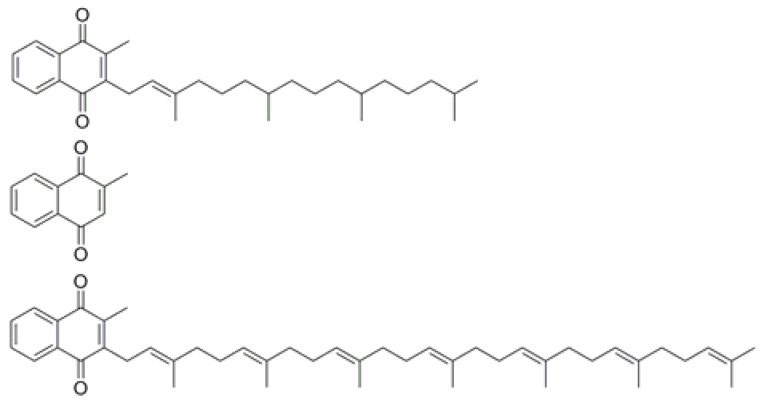
Chemical structure of Vitamin K_1_ (upper figure), the synthetic analogue vitamin K_3_ (middle), and MK-7 (bottom).

Stabilizers were removed from EGDMA and MAA by passing it over aluminum oxide. Due to its bulky, aliphatic, flexible side chain (Figure 1), phylloquinone (vitamin K_1_) is not suitable for imprinting. Therefore, menadione (vitamin K_3_) was used as a template in a dummy imprinting approach. This “synthetic vitamin K” has a structure similar to the head group of vitamin K_1_ but it lacks the aliphatic chain. Vitamin K_3_ MIPs were synthesized by dissolving the functional monomer MAA (1.74 mmol), cross-linker molecule EGDM (2.90 mmol), and initiator azobisisobutyronitrile (AIBN) (50 mg) in dimethyl sulfoxide (3 mL) together with the menadione (50 mg, 0.29 mmol). The mixture was purged for 5 min with nitrogen gas to remove any oxygen or water from the mixture. Polymerization was thermally initiated at 65 °C and the polymer was left to cross-link for 12 h.

Non-Imprinted Polymers (NIPs), serving as a reference, were made using the same protocol without including the target to the pre-polymerization mixture. MIPs and NIPs were milled seven times using a Fritsch Planetary Micro Mill Pulverisette 7 premium line (Idar-Oberstein, Germany) (700 rpm, 5 min, 10 mm balls) After milling, the particles were sieved at 1.0 mm amplitude using a Fritsch Analysette 3 for 4 h or until sufficient amount of polymer was on the collection plate and the 20 μm sieve. The particles collected were between 20–45 μm. Particle size was confirmed using a Scanning Electron Microscope (SEM, Philips XL 30Eindhoven, The Netherlands analysis shown in Supplementary Materials Figure S1). MIPs were extracted using a Soxhlet extraction procedure, in which MIPs were washed for 48 h with a 9:1 mixture of methanol and acetic acid, followed by a final extraction step with methanol during 24 h. The polymers were dried at 65 °C for 72 h prior to immobilizing them onto measurement chips.

### 2.3. Chip Preparation

Polished aluminum plates were cut to obtain chips with the desired dimensions (10 × 10 mm^2^). To immobilize MIP particles, a 100 nm polyvinyl chloride (PVC) adhesive layer (0.35 wt % PVC dissolved in tetrahydrofuran) was deposited on the chip by spin coating at 3000 rpm for 60 s with an acceleration of 1100 rpm/s. MIP and NIP particles were stamped into this layer using a PDMS substrate that was covered with a monolayer of polymer particles. The PVC layer was heated for 2 h at a temperature above its glass transition temperature (100 °C) allowing the beads to sink into the polymer layer. The samples were cooled down prior to thermal measurements and any unbound particles were washed off with distilled water.

### 2.4. Sensing Setup

The thermal detection platform is described thoroughly in previous work [31,32,33]. Functionalized chips were pressed mechanically with their backside onto a copper block serving as a heat provider. The temperature of the copper underneath the sample, T_1_, was monitored by a K-type thermocouple (TC Direct). This information was fed into a temperature control unit that stringently controlled T_1_ by modifying the voltage over the power resistor (Farnell, Utrecht, The Netherlands) that heats the copper, using a software-based (Labview, National Instruments, Austin, TX, United States) Proportional-Integral-Derivative (PID) controller (P = 10, I = 5, D = 0). The functionalized side of the chip faced a PolyEther Ether Ketone (PEEK) flow cell which was sealed with an O-ring to avoid leakage, defining a contact area of 28 mm^2^ and an inner volume of 110 µL. The flow cell is connected to a tubing system, allowing to exchange liquids in a controlled and automated fashion by means of a syringe pump. The temperature of the liquid inside the flow cell, T_2_, is measured by a second thermocouple placed 1 mm above the chip. 

For each rebinding measurement, the signal was stabilized in acetonitrile which was used as a background solvent for the measurements, seeing as vitamin K does not dissolve in water. The concentration of the target or analogue inside the flow was gradually increased (50 nM–2.25 µM). The signal was allowed to stabilize for 30 min between subsequent additions. Data were analyzed by monitoring the decrease in T_2_ after each addition (heat transfer method or HTM) while maintaining T_1_ at a constant 37.00 °C. At the end of each stabilization platform, a thermal wave was sent through the chip with an amplitude at 0.1 °C and a variable frequency that increased from 0.01 to 0.05 Hz. The transmitted wave was recorded and compared to the transmitted wave at baseline in terms of the observed phase shift and decrease in amplitude (Thermal Wave Transport Analysis, or TWTA).

### 2.5. Proof of Concept

To assess the sensor’s potential for application in biochemical studies, an experiment was designed to verify whether it was possible to determine the difference between an extracted serum sample before and after vitamin K intake. Fasting blood was drawn from one of the authors in the morning to have a baseline condition for vitamin K. Following this, 5 mg of MK-7 (menaquinone-7; kind gift of Nattopharma ASA, Hovik, Norway) was taken together with a breakfast low in vitamin K_1_ and K_2_, and blood was drawn 5 h after ingestion (known to be the peak absorption [50]). The serum was prepared and extracted with hexane and methanol. The water and methanol phase contains the water-soluble components and precipitated proteins. The hexane phase containing vitamin K was removed and evaporated under a constant stream of nitrogen at 37 °C, and the residue was dissolved in hexane. The hexane sample was divided and measured by TWTA and on conventional Reversed Phase (RP) HPLC. The study was conducted in accordance with the Declaration of Helsinki, and the protocol was approved by the Ethics Committee of Maastricht University Medical Center+.

The RP-HPLC detection method, based on fluorimetric detection after post-column zinc reduction, was introduced in previous work [50]. Briefly, after adding an internal standard (Vitamin K_1_(25); a synthetic form of vitamin K_1_ containing 5 isoprenoid residues) to the sample, vitamin K was purified over Sep-pak cartridges and eluted from the silica with ether-hexane (3:97, *v*/*v*). The fraction containing vitamin K was concentrated by evaporating the hexane and reconstitute the residue with 0.2 mL isopropanol which was injected.

## 3. Results and Discussion

### 3.1. Specific Detection of Vitamin K_3_

**Figure 2:**
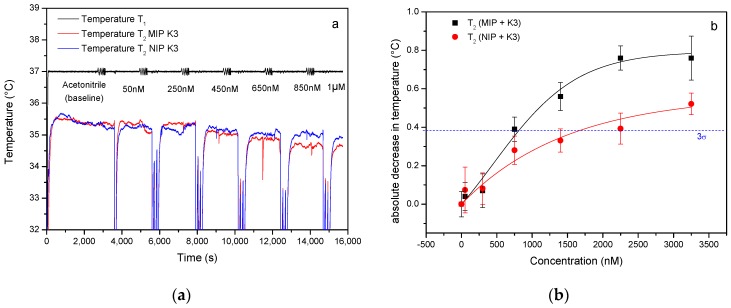
Heat-Transfer Method (HTM) analysis of a vitamin K_3_ rebinding experiment on Molecularly Imprinted Polymers (MIP) and a non-imprinted reference. (**a**) The time-dependent temperature profile shows that starting from a total concentration of 700 nM, the temperature inside the flow cell decreases as more vitamin K_3_ is bound to the MIP. As the concentration of the target increases, the effect becomes more pronounced. (**b**) The dose-response curve reveals that the limit-of-detection (illustrated by the blue dotted 3 sigma line) is ±700 nM. Non-imprinted polymer: NIP.

The HTM analysis of an experiment in which both a MIP-coated chip and a NIP-coated reference (Figure 2a) are exposed to vitamin K_3_ illustrate that the temperature inside the flow cell decreases with an increasing menadione concentration. These findings are in line with previously obtained results using MIP-based thermal sensors for the detection of neurotransmitters [31]. Binding the target into the binding cavities of the polymer particles increases the thermal resistance of the solid-liquid interface, impairing thermal transport into the flow cell. The data were used to construct a dose-response curve (Figure 2b). As the inter-sample variability on measurements done using three different samples from different batches was lower than the noise on the signal, the latter was used to construct the error bars. Both the error bars and average temperature were calculated over a period of 1000 s. The data were fit in OriginPro 8 (OriginLabs Corporation, Northampton, MA, United States) using a dose-response fit for both the MIP (black curve, *R*^2^ = 0.9931) and NIP electrode (red curve, *R*^2^ = 0.9541). The blue dashed line indicates the value that corresponds to three times the maximum amount of noise on the signal throughout the measurement and its intercept with the linear part of the black curve predicts a preliminary Limit of Detection (LoD) of 700 nM (3σ method). 

**Figure 3:**
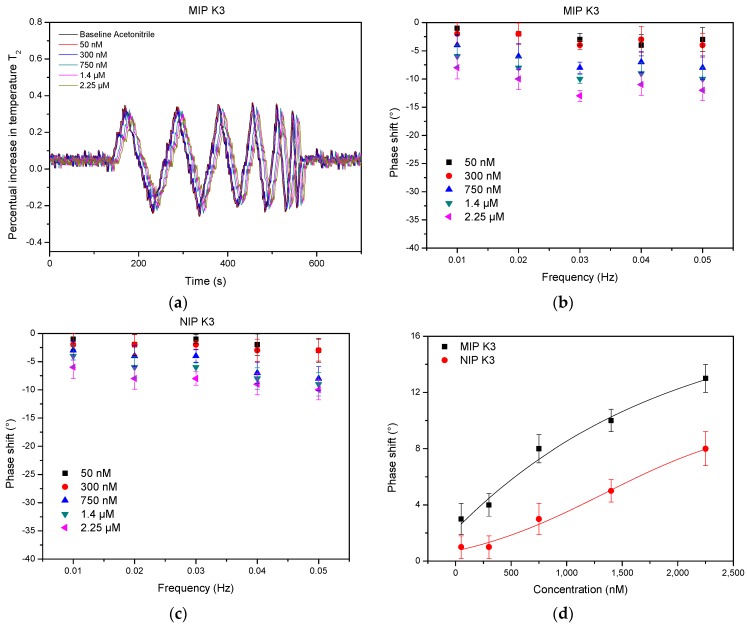
TWTA analysis of a vitamin K_3_ rebinding experiment on a MIP-coated electrode and a non-imprinted reference. (**a**) A delay on the transmitted wave and decrease in amplitude can be observed that increases as the concentration of target inside the flow cell increases. (**b**) The Bode plot indicates that the best concentration-dependent resolution is obtained at 0.03 Hz. (**c**) The bode plot of the NIP shows a similar behavior but the effect size is lower. (**d**) The dose-response curve reveals that the limit of detection (illustrated by the blue dotted 3 sigma line) is in the 200 nM range.

In addition to HTM, the data were also analyzed using the TWTA method that has proven to be faster and more sensitive in previous research [33]. The results of this analysis (Figure 3a) show that the transmitted wave experiences a concentration-dependent phase shift. The increase in thermal resistance and thermal mass on the surface of the chip leads to an impaired transmission of the thermal wave, which can be observed as a phase shift and a decrease in the wave amplitude. The thermal wave Bode plot was calculated over a period of a 1000 s and reveals that an optimal resolution and effect size can be achieved at 0.03 Hz (Figure 3b), which confirms previous findings [33]. The Bode plot of the TWTA analysis using a NIP electrode (Figure 3c, wave analysis in Supplementary Materials Figure S2) reveals a similar behavior, but an imprinting factor between 1.7 and 2 can be observed over the entire concentration range, indicating that a significant part of the signal can be attributed to specific recognition of the target. The obtained data were used to construct a dose-response curve (Figure 3d) that corresponds well with the allometric dose-response fit that was applied for both the MIP (black curve, *R*^2^ = 0.93711) and the NIP electrode (red curve, *R*^2^ = 0.99685). The LoD was calculated using the 3σ method and was established at 200 nM. This improved sensitivity can be attributed to the fact that the noise on the transmitted wave in TWTA is much less pronounced. These findings are in line with results that were previously obtained for bacteria and dopamine detection [29,33]. All measurements were validated using a traditional batch rebinding experiment that is summarized in Supplementary Materials Figure S3. 

### 3.2. Vitamin K_1_ Detection

**Figure 4:**
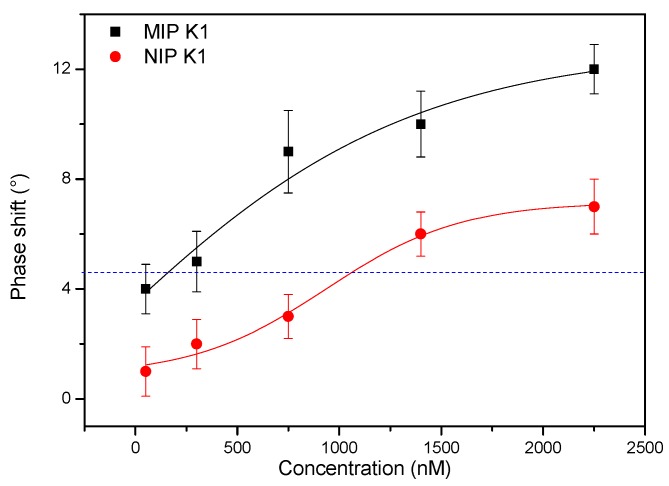
TWTA analysis of both a MIP, imprinted for vitamin K_3_, and NIP electrode exposed to an increasing concentration of vitamin K_1_ in acetonitrile. A concentration-dependent increase can be observed for both MIP (black curve) and NIP, but the imprinting factor varies between 1.5 and 2 over the entire concentration range. The dashed line represents the value corresponding to three times the maximal error on the signal. Its intercept with the dose-response fit for the MIP electrode defines a limit of detection in the range of 200 nM.

To test the hypothesis that MIPs imprinted with menadione should also be able to detect the natural vitamin K_1_, vitamin K_3_ MIPs and NIPs were immobilized onto aluminum chips and exposed to an increasing concentration of vitamin K_1_ in acetonitrile. The TWTA analysis (HTM analysis, wave spectrum, and Bode plots can be found in Supplementary materials Figure S4) reveals a dose-response curve at 0.03 Hz for vitamin K_1_, which is similar to that obtained for the template vitamin K_3_ (Figure 4). 

A concentration-dependent increase in phase shift is observed for both MIP and NIP over the entire concentration range, with imprinting factors varying from 1.5 to 2. Although the maximum error on the signal for the vitamin K_1_ measurements was slightly higher, resulting in a higher 3σ value, the LoD was also established to be in the 200 nM range. These findings confirm the hypothesis that imprints made by vitamin K_3_ and vitamin K_1_ are identical as the head group of vitamin K is mainly responsible for the imprinting effect. The similar effect size observed in this experiment in comparison to the data in Figure 3 can be considered as somewhat surprising, seeing as vitamin K_1_ can only bind to the surface imprints on the MIP particles. However, the thermal resistance effects are mainly affected by changes happening at the surface boundaries that form the solid–liquid interface. In addition, binding the bulky Vitamin K_1_ to the receptor layer appears to increase both the thermal resistance and thermal mass at the solid–liquid interface to a larger extent than the smaller menadione molecule, compensating for the fact that it can only bind to the surface of the MIP particles. 

### 3.3. Selectivity Test

The selectivity of the sensor was examined by exposing vitamin K_3_-imprinted electrodes to increasing concentrations of the analogue vitamin E (γ-tocopherol) in acetonitrile, in a manner similar to the measurements described in the previous section, and comparing the response to that obtained with vitamin K. The HTM and TWTA analysis as well as the Bode plot can be found in the Supplementary Materials Figure S5. The TWTA response at 0.03 Hz was used to construct a dose-response curve that was compared to the dose-response curve for the MIP and NIP exposed to vitamin K_1_ (Supplementary Materials Figure S6). The data show that phase shift increases in function of the vitamin E concentration in the measuring cell. The data can be fitted using an allometric dose-response function (*R*^2^ = 0.99955) and demonstrate that most of the vitamin E binds in a non-specific manner to the functional interface as the response (blue curve) is nearly identical to that of an electrode coated with NIP particles (red curve) whereas the reaction of a MIP towards the target, vitamin K_1_, is a lot more pronounced (black curve). These findings illustrate that although the structure and size of γ-tocopherol and vitamin K_1_ are comparable, there is no specific interaction between the analogue and the binding sites created within the MIP particles. This further confirms the hypothesis that the head group of the template interacts with the forming polymer during formation and results in the formation of binding cavities that are able to selectively rebind vitamin K.

### 3.4. Proof of Application

**Figure 5:**
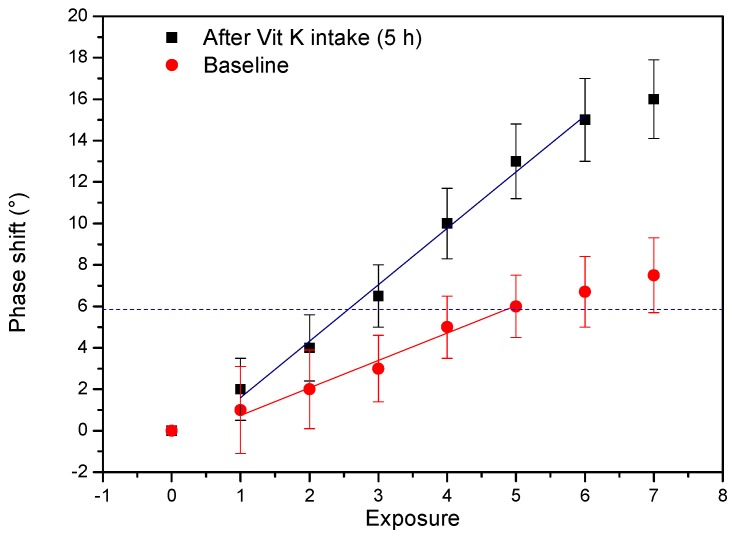
TWTA analysis of a blood serum sample before (baseline, red curve) and after ingestion of MK-7 tablets (black curve). A progressive enrichment strategy was applied for the 5 h sample that has an increasing effect on the signal with each exposure run. The blue line indicates the demarcation for a relevant increase in signal that is barely reached for the baseline, whereas a relevant concentration can be determined for the five-hour sample after three exposure runs and more. A linear fit was applied to determine the original concentration in the sample.

The hexane-extracted samples were analyzed using the MIP-based TWTA sensor system (Figure 5). Due to the low concentration of vitamin K, a gradual enrichment strategy was employed. The MIP layer was exposed seven consecutive times to the same 3 mL sample by modifying the pumping system and creating a closed-circuit loop. As expected, the signal gradually increases for both the baseline and the five-hour sample. After three exposure runs, a significant phase shift (3σ method) of 6.5 ± 1.1° can be observed for the five-hour sample. This falls within the relevant, linear range of the calibration curve and corresponds to a concentration of 593 ± 80 nM, as calibrated from the MK-7 calibration curve shown in Supplementary Materials Figure S7. To calculate the original concentration, the linear part of the graph in Figure 5 was fitted, leading to a value of 1.595 ± 1.1° for the first exposure run, which corresponds to 145 ± 80 nM. The same can be done for the baseline, although the signal barely reaches the significant level after five runs. The Vitamin K concentration MK-7 and vitamin K_1_ in the baseline sample was analogously determined at 67 ± 80 nM. The hexane samples were analyzed using HPLC, resulting in a total vitamin K content of 70.4 nM in the sample that was taken 5 h after ingestion of MK-7, which corresponds well with the value obtained with the sensor. The baseline was determined at 3.3 nM and therefore diverges from the sensor measurement by an order of magnitude. This can be explained by the fairly large error on the methodology in its present form. Even more interesting is the difference between the black and the red curve in Figure 5, which corresponds to a value of 64 nM and should be caused by MK-7 intake. The HPLC was able to determine the MK-7 content in the hexane sample specifically and registered a value of 69 nM for the five-hour sample and a concentration of 1.9 nM in the baseline sample, corresponding to a difference of 67.1 nM. This striking agreement illustrates the potential use of TWTA in biochemical assays in the future, but must be looked at critically, since the error on the measurement is large. 

## 4. Conclusions

Vitamin K is an essential dietary micro-nutrient, needed as an unequivocal cofactor in the activation of so-called vitamin K dependent proteins. With the discovery that vitamin K dependent proteins are involved in the inhibition of ectopic calcification, vitamin K has gained interest from both society and clinicians [51]. Traditional tests for assessing vitamin K concentrations include reversed-phase HPLC and mass spectrometry. These tests are expensive, labor-intensive, and time-consuming. This article describes the development of a MIP-based thermal biomimetic sensor that is able to selectively distinguish vitamin K_1_ and vitamin K_2_ (MK-7) from other vitamins using MIPs imprinted with the synthetic vitamin K analogue menadione. A proof of principle was established in this manner, demonstrating an MIP-based identification of vitamin K in acetonitrile. A preliminary limit of detection of 200 nM was established for TWTA, whereas the original HTM appears to be a little less sensitive, which is in line with findings from previous studies. Although the sensitivity and selectivity of the synthetic receptor layers can still be optimized in the future, the gradual enrichment approach illustrates that it is possible to use the sensor to study the vitamin K content of blood serum samples with little sample pre-treatment needed. Improving the sensitivity of the platform by optimizing MIP synthesis and deposition and improving the sensor design to bring down the error on the measurement are both important next steps. It is however still necessary to study small physiological differences in vitamin K content that typically occur in the low nanomolar range. 

## Supplementary Materials

The following are available online at http://www.mdpi.com/2072-6643/10/6/751/s1, Figure S1: SEM analysis of MIP particles, Figure S2: HTM analysis on both a MIP and a NIP electrode that are exposed to an increasing concentration of vitamin K_1_, Figure S3: UV-Vis batch rebinding analysis, Figure S4: TWTA analysis on both a MIP and a NIP electrode that are exposed to an increasing concentration of vitamin K_1_, Figure S5: HTM and TWTA analysis on both a MIP and a NIP electrode that are exposed to an increasing concentration of vitamin K_1_ and vitamin E, Figure S6: TWTA analysis on a MIP exposed to an increasing concentration of vitamin E. (a) Wave analysis. (b) Bode plot, Figure S7: calibration curve for MK-7.
