# Supplementary material for: A Novel Biomimetic Tool for Assessing Vitamin K Status Based on Molecularly Imprinted Polymers
Source: Nutrients. 2018 Jun 11;10(6):751. doi: 10.3390/nu10060751 (PMC6024727; doi:10.3390/nu10060751)
Supplement: Supplementary file 1 [file nutrients-10-00751-s001.pdf]

nutrients

Supplementary Materials

A Novel Biomimetic Tool for Assessing Vitamin K Status Based on Molecularly Imprinted Polymers

Kasper Eersels [1], Hanne Diliën [1], Joseph W. Lowdon [1], Erik Steen Redeker [1], Renato Rogosic [1], Benjamin Heidt [1], Marloes Peeters [2], Peter Cornelis [3], Petra Lux [4], Chris P. Reutelingsperger [4], Leon J. Schurgers [4], Thomas J. Cleij [1] and Bart van Grinsven [1,]*

[1] Maastricht Science Programme, Faculty of Science and Engineering, Maastricht University P.O. Box 616, 6200 MD Maastricht, The Netherlands; kasper.eersels@maastrichtuniversity.nl (K.E.); hanne.dilien@maastrichtuniversity.nl (H.D.); joe.lowdon@maastrichtuniversity.nl (J.L.); erik.steenredeker@maastrichtuniversity.nl (E.S.R.); renato.rogosic@maastrichtuniversity.nl (R.R.); benjamin.heidt@maastrichtuniversity.nl (B.H.); thomas.cleij@maastrichtuniversity.nl (T.J.C.)

[2] Division of Chemistry and Environmental Science, School of Science and the Environment, Faculty of Science and Engineering, Manchester Metropolitan University, Chester Street, Manchester M1 5GD, UK; m.peeters@mmu.ac.uk

[3] Soft-Matter Physics and Biophysics Section, Department of Physics and Astronomy, KU Leuven, Celestijnenlaan 200 D, B-3001 Leuven, Belgium; peter.cornelis@kuleuven.be

[4] Department of Biochemistry, Cardiovascular Research Institute Maastricht, Universiteitssingel 50, 6200 MD Maastricht, The Netherlands; p.lux@maastrichtuniversity.nl (P.L.); c.reutelingsperger@maastrichtuniversity.nl (C.P.R.); l.schurgers@maastrichtuniversity.nl (L.J.S.)

* Correspondence: bart.vangrinsven@maastrichtuniversity.nl; Tel.: +31-(0)43-3882282

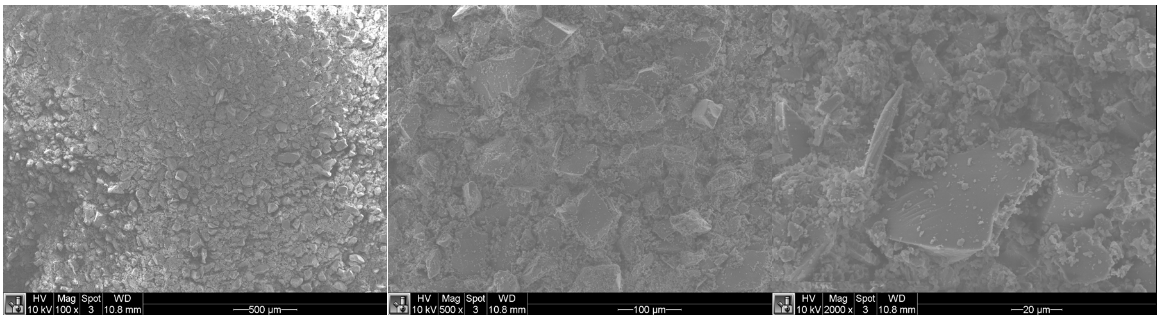

Figure S1. SEM analysis of MIP morphology. Images were taken at magnifications of 100, 500 and 2000 to show the general morphology and size distribution of the MIP particles. The observed heterogeneous morphology of the particles is typical for bulk polymerization.

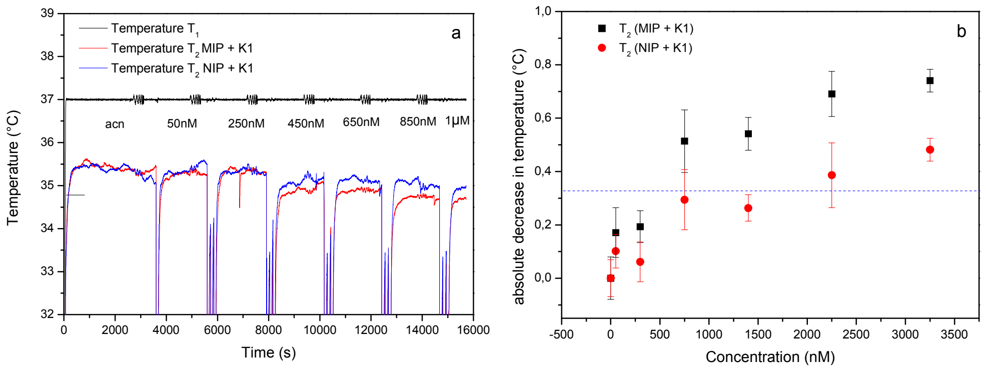

Figure S2: HTM analysis on both a MIP and a NIP electrode that are exposed to an increasing concentration of vitamin K1. (a) Time-dependent temperature profile. (b) dose-response curve.

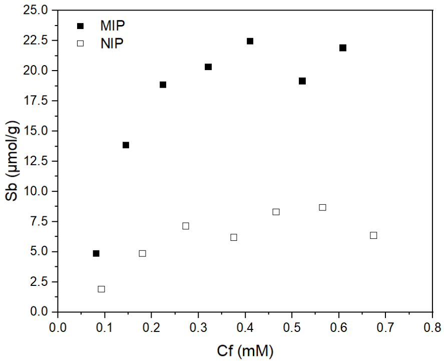

Figure S3. Result of a batch-rebinding study using UV-Vis spectroscopy. A vitamin K3 imprinted MIP and a non-imprinted reference were exposed to increasing concentrations of the target. The results show that the MIP is able to specifically detect the target.

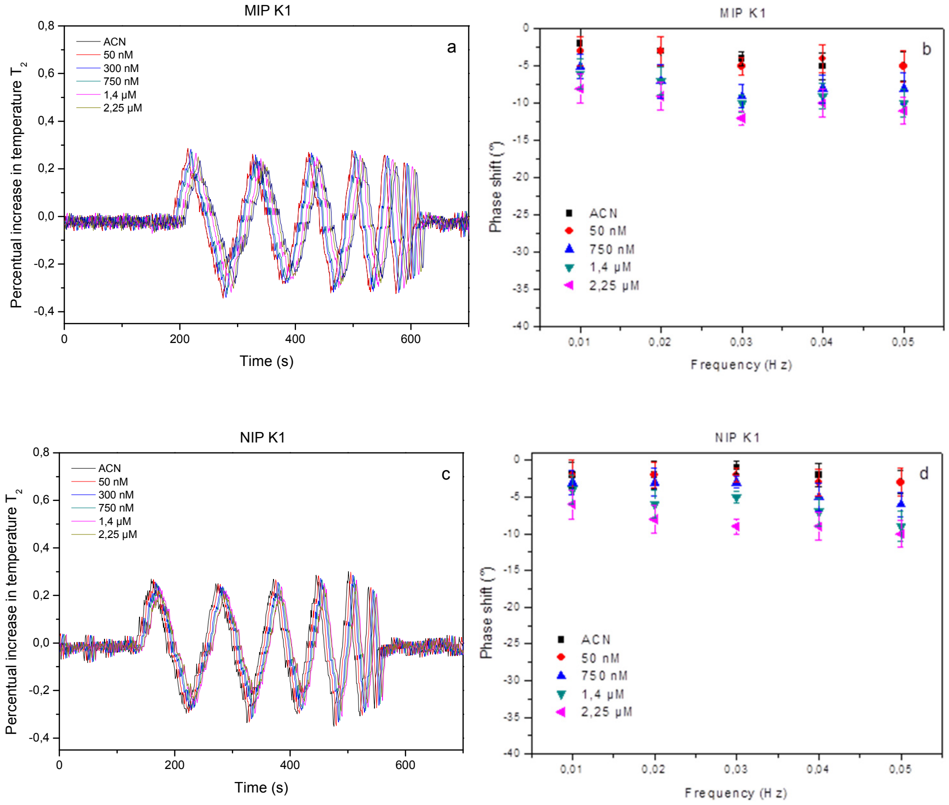

Figure S4: TWTA analysis on both a MIP and a NIP electrode that are exposed to an increasing concentration of vitamin K1. (a) Wave analysis MIP. (b) Bode plot MIP. (c) Wave analysis NIP. (d) Bode plot NIP.

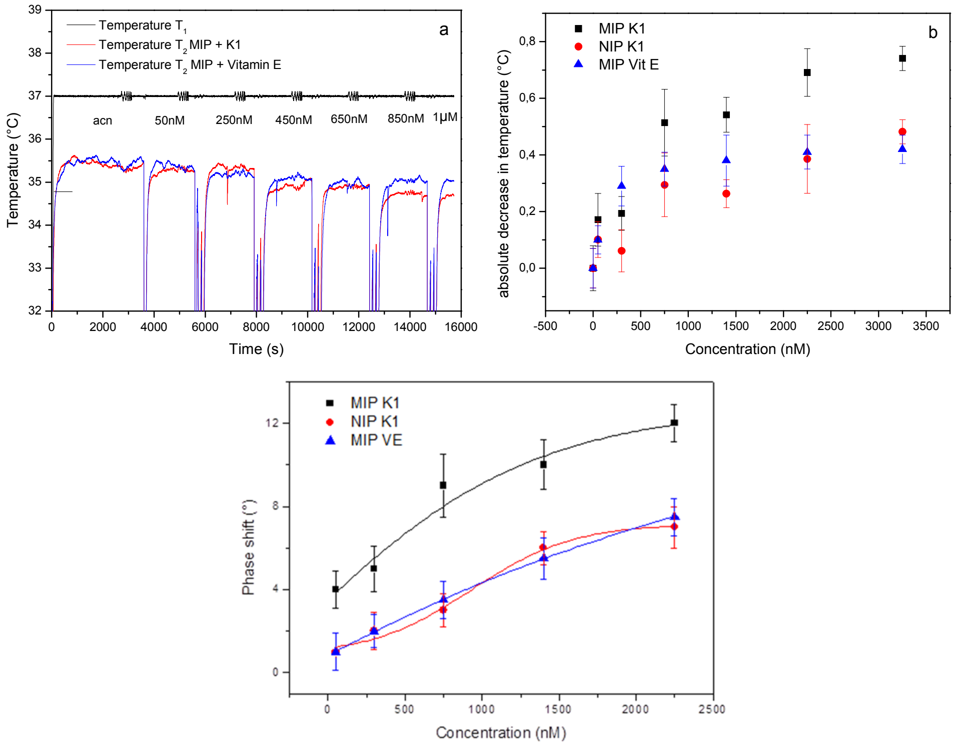

Figure S5: HTM analysis on both a MIP and a NIP electrode that are exposed to an increasing concentration of vitamin K1 and vitamin E. (a) Temperature profile. (b) Dose-response curve. (c) Fitted dose-response curve.

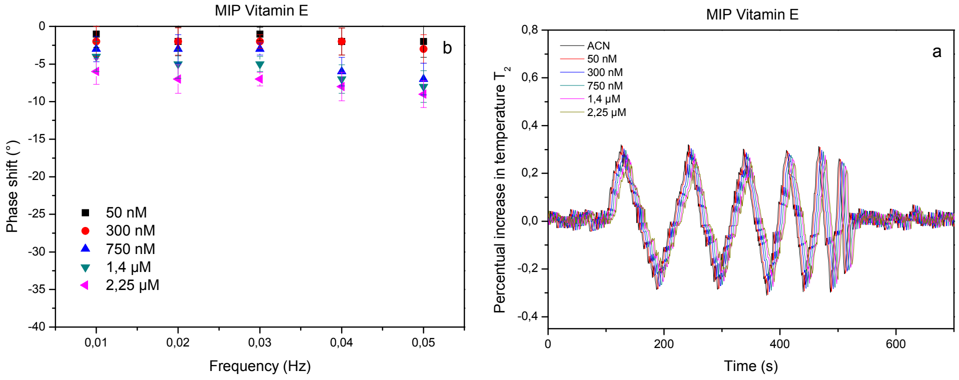

Figure S6: TWTA analysis on a MIP exposed to an increasing concentration of vitamin E. (a) Wave analysis. (b) Bode plot.

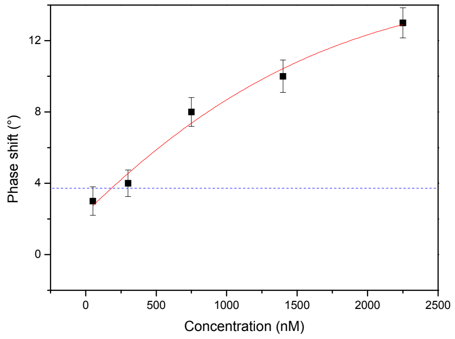

Figure S7: Calibration curve for a menadione-MIP exposed to an increasing concentration of MK-7.
